# Spatial-temporal risk assessment of urbanization impacts on ecosystem services based on pressure-status - response framework

**DOI:** 10.1038/s41598-019-52719-z

**Published:** 2019-11-14

**Authors:** Peng Kang, Weiping Chen, Ying Hou, Yuanzheng Li

**Affiliations:** 1 State Key Laboratory of Urban and Regional Ecology, Research Center for Eco-Environmental Sciences, Chinese Academy of Sciences, Beijing, 100085 P.R. China; 2 University of Chinese Academy of Sciences, Beijing, 100049 P.R. China

**Keywords:** Ecosystem services, Urban ecology

## Abstract

Rapid urbanization is a global phenomenon that has altered many ecosystems, generating ecological risks such as causing a decline in many ecosystem services. In this study, ecosystem service oriented risk assessment combined with PSR were quantifying how urbanization influences the ecosystem services about Beijing-Tianjin-Hebei urban agglomeration of China between 2000 year and 2010 year. The mean value of ecosystem services in three gradient (rural areas, suburban, and urban area) declined from 4.12 Yuan/m^2^ to 1.75 Yuan/m^2^ in 2000 year, while the mean value in 2010 year showed significant decrease and also represented urban-rural gradient. The average of pressure in PSR framework increased from 0.145 to 0.162 between two periods, while the average of status decrease from 0.378 to 0.311, and the status value decrease from 0.096 to 0.087. The higher risk degree V increased 6.95% between two periods, while the lower risk degree I decrease 6.89%. Two main types including high value gathering field and low value gathering field existed between two periods, the higher gathering field owned the ratio of 9.85%, mainly distributed around the urban area of Beijing and Tianjin, while the lower gathering field possess the ratio of 10.69%, mainly distributed in the northern and western in region. Overall, the analytical framework proposed in this study can provide comprehensive information to evaluate the impacts of complex practice in land-use planning and region ecosystem management.

## Introduction

As population concentrations grew and economic activities intensified, the demand for developed land (e.g., housing, infrastructure, and business centers) increased, and the consequent growth in regional areas can be regarded as urbanization^1,2^. People dwelling in urban areas now account for almost 50% of the world’s population, and the prospect is that the urbanization ratio will reach 60% by 2030^3,4^. Rapid urbanization has resulted in many ecological problems worldwide, which has changed the interactions among the atmosphere, hydrosphere, and biosphere, consequently, altering many ecosystem services, resulting in their broad decline^5–7^.

Assessment the impact of land-use change on ecosystem service is one of the critical endeavors in regional management and become the hotspots of regional sustainable development^8–10^. Such assessments help identify which services are declining because of urbanization^11,12^. Many studies have examined how urban–rural spatial gradients influence ecosystem services^13–15^. Other studies have examined the spatial-temporal changing influence of urbanization on ecosystem services over time^16–18^. As a whole, it is trying to maintain a balance between environmental sustainability and continuing urbanization^19,20^. However, considering the complex interaction in regional ecosystem, it is unreasonable to paid main attentions for ecosystem services but to ignore the related ecological process and function.

The ecological risks generated by urbanization are major contributors of global change^21^.The extension of artificial surface has resulted significant alterations in the structure and processes of ecosystems, which are consequently impaired in their capacity to deliver the expected services^22,23^. A few initiatives have been developed to detect ecological risks generated by urbanization for acquiring quantitative knowledge about how urbanization processes influence ecosystem services^24,25^. Ecosystem service-oriented risk assessment framework not only strengthen the linkage between ecological processes and ecological risk sources, but also provide quantitative information to initiate sustainable ecosystem management^26,27^. However, theoretical and analytical frameworks should need further advancement to establish the logical relationship among risk sources, ecological components and ecosystem service, and to take them as an integral system rather than focusing on individual ES^28–30^.

The main objective of this research is to propose and test a comprehensive framework for assessing the ecosystem services influenced by artificial surface, which integrates ecological process and function, ecological risk domains and spatial analysis in regional ecosystems. Specifically, we aimed to: (1) develop a risk assessment framework oriented to ecosystem service coupling with PSR (pressure-states-response) structure; (2) discern the spatial pattern and urban-rural gradient of ecological index and ecosystem services in regional ecosystems under influences of rapid urbanization; (3) analyze spatial and temporal interactions between ecosystem services and the extension of artificial surface by using risk valuation methods. Beijing-Tianjin-Hebei urban agglomeration is selected as a case which has experienced a fast increase of impervious surface and presents a typical urban-rural gradient.

## Result

### Urban-rural gradient of ecological index and ecosystem service

**Figure 1:**
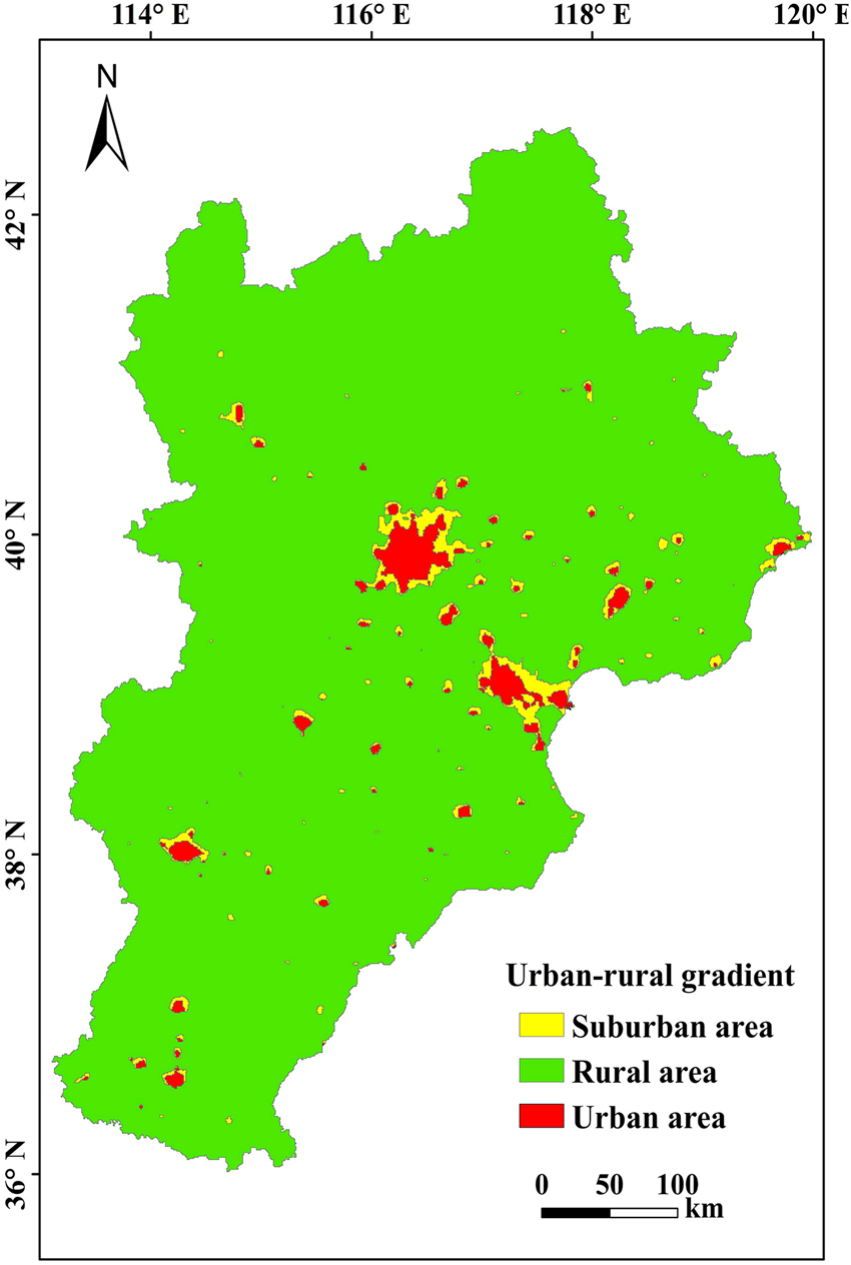
The classification of urban-rural gradient. Map generated in ArcGIS 10.2.

The classification of urbanization was depicted in Fig. 1. The urban area is 4330 km^2^, which mainly distributed in the central and eastern part, and other parts scattered in the southern region. The suburban area is 4786 km^2^, that basically surrounded about urban field, that indicated that artificial surface increasingly extended, especially happened in the urban developed field of Beijing and Tianjin. Finally the rural field covered the entire region, accounting for most of the region.

**Figure 2:**
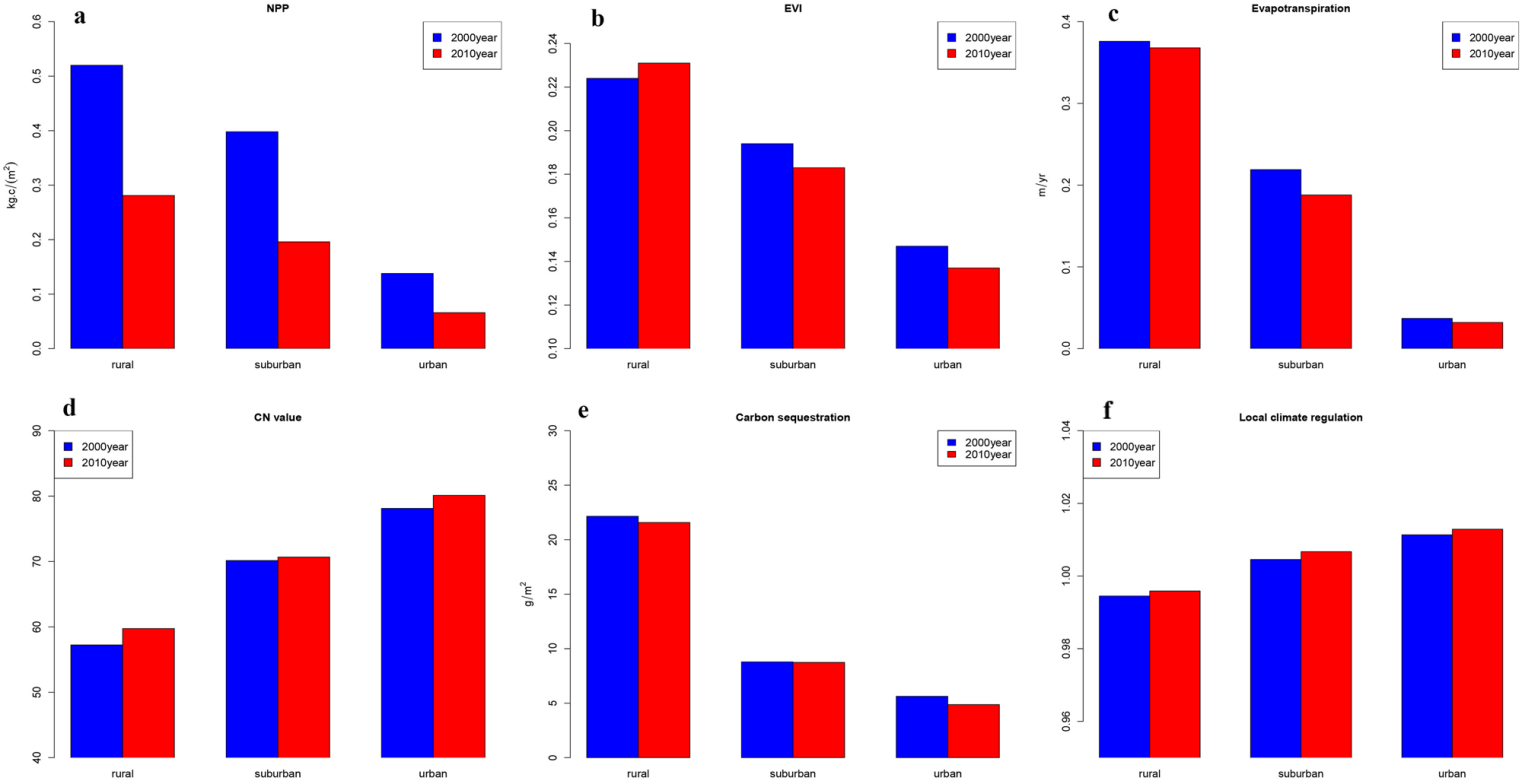
The urban-rural gradient of ecological index. (**a**) Urban-rural gradient about net primary production. (**b**) Urban-rural gradient about EVI. (**c**) Urban-rural gradient about evapotranspiration. (**d**) Urban-rural gradient about curve number. (**e**) Urban-rural gradient about carbon sequestration. (**f**) Urban-rural gradient about local climate regulation. Figures were generated with R langauge program 3.1.3.

The average values of the six indicators along the urban-rural gradient were shown by Fig. 2. The mean values of net primary production for the three gradient areas in 2000 year decreased from 0.52 kg.c/m^2^ to 0.12 kg.c/m^2^, whereas the mean values in 2010 year also showed a significant urban-rural gradient, but the value was only half of those in 2000 year. In terms of EVI, the mean values of three gradient areas changed from 0.225 to 0.14 in 2000 year. For the year 2010, the mean values of EVI of the rural field was less than that of 2000, but the value of other fields was significantly more than those of 2000. For the evapotranspiration, the mean values of the three gradient areas in 2000 year decreased from 0.38 m/yr to 0.03 m/yr, and the values in 2010 was similar to those in 2000 but a little decline. The mean values of CN for the three gradient areas in 2000 year increased from 58 to 78; while the value in 2010 year was obviously higher than the value of 2000 year especially in rural and urban region. In term of carbon sequestration, the mean value in rural is significantly higher than two other regions. The mean value of three gradient in 2000 year decreased from 22 g/m^2^ to 6 g/m^2^; while the mean value in 2010 year was slightly lower than 2000’s, except for urban field. The local climate regulation also showed a urban-rural gradient increasing from 0.99 to 1.02, and the gap between two years was small.

**Figure 3:**
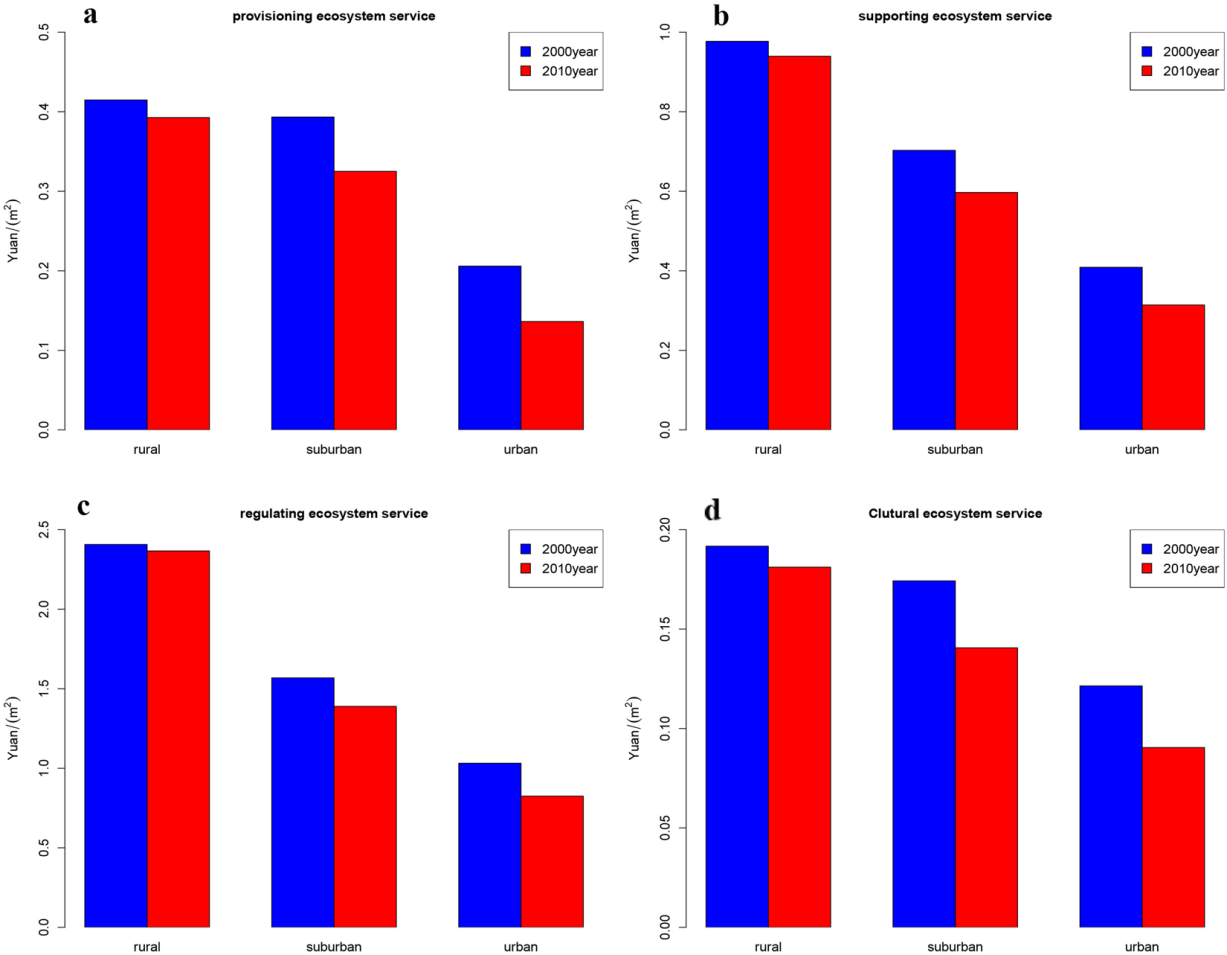
The urban-rural gradient of ecosystem services. (**a**) Urban-rural gradient about provisioning ecosystem service. (**b**) Urban-rural gradient about supporting ecosystem service. (**c**) Urban-rural gradient about regulating ecosystem service. (**d**) Urban-rural gradient about cultural ecosystem service. Figures were generated with R langauge program 3.1.3.

The average values of four type of ecosystem service along the urban-rural gradient were shown by Fig. 3. For the providing ecosystem service, the mean value of rural and suburban field was obviously higher than the mean value of urban area. The mean value of three gradient declined from 0.42 Yuan/m^2^ to 0.22 Yuan/m^2^, while mean value of 2010 year was to some extent lower than 2000’s, especially in urban area. In term of supporting ecosystem service, the mean value of three gradient in 2000 year decreased from 1.0 Yuan/m^2^ to 0.4 Yuan/m^2^. The mean value of 2010 year was significant lower than the mean value of 2000 year, especially in urban field or suburban field. In term of regulating service, the mean value of rural was obviously higher than the other two regions. The mean value of three gradient in 2000 year decreased from 2.5 Yuan/m^2^ to 1.0 Yuan/m^2^, while the mean value of 2010 year was much lower than the mean value of 2000 year except for rural field. For the cultural ecosystem service, the mean value of rural and suburban field was obviously higher than the mean value of developed urban area, declining from 0.19 Yuan/m^2^ to 0.13 Yuan/m^2^ in 2000, and from 0.18 Yuan/m^2^ to 0.09 Yuan/m^2^ in 2010.

Significant differences were ascertained among four type of ecosystem services (providing service; supporting service; regulating service; culture service) along the rural–urban gradient, particularly in urban environments where the values of these services were significantly lower than in rural and suburban area. Therefore, artificial surface ratio can be viewed as the predominant factor for rural-urban gradient which plays a key role in the distribution of ecosystem services.

### Changes of three components of PSR framework

**Figure 4:**
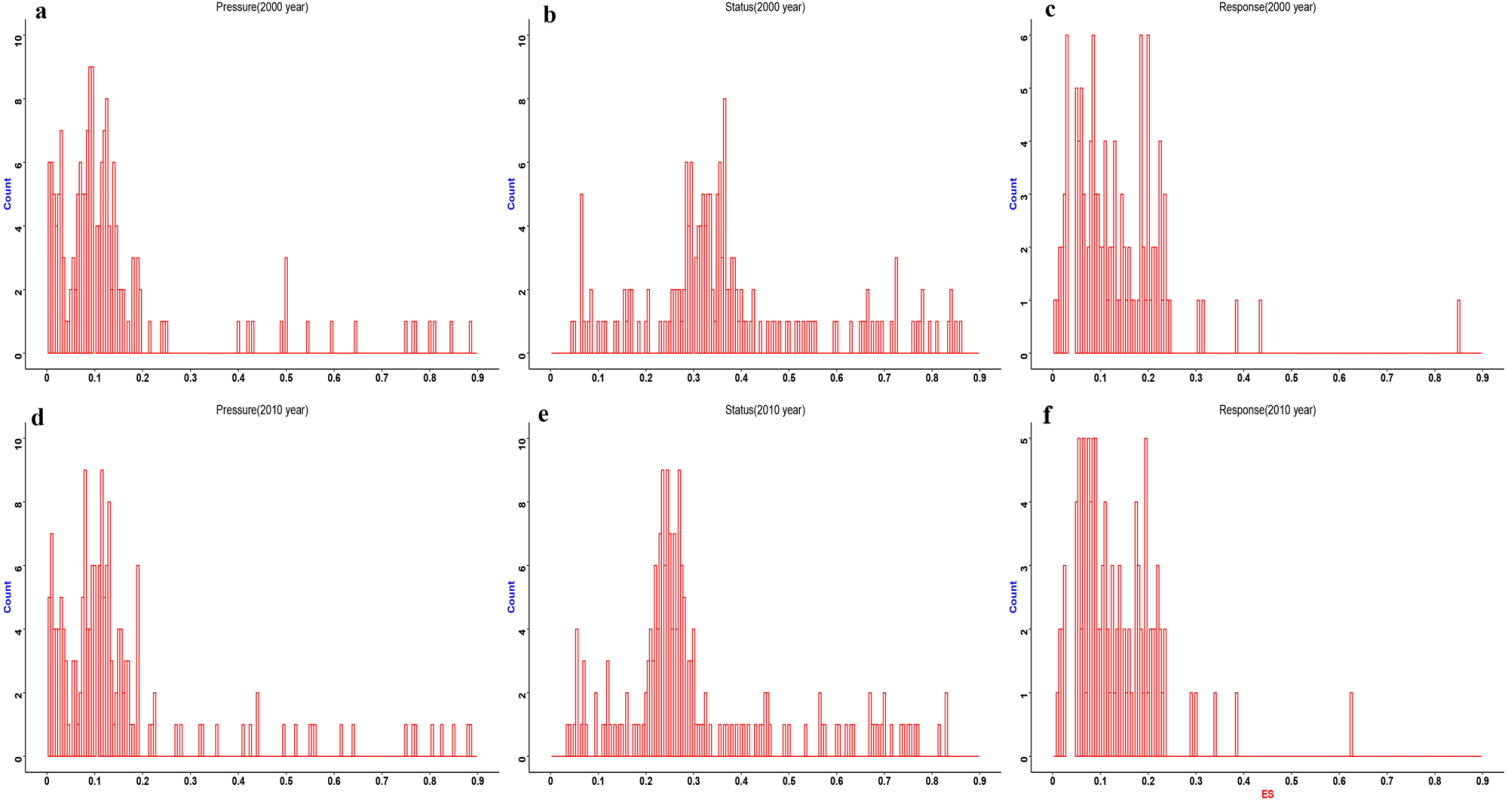
The histogram about three components about PSR framework in 2000 and 2010. (**a**) histogram about pressure in 2000. (**b**) Histogram about status in 2000. (**c**) Histogram about response in 2000. (**d**) Histogram about pressure in 2010. (**e**) Histogram about status in 2010. (**f**) Histogram about response in 2010. Figures were generated with R langauge program 3.1.3.

**Figure 5:**
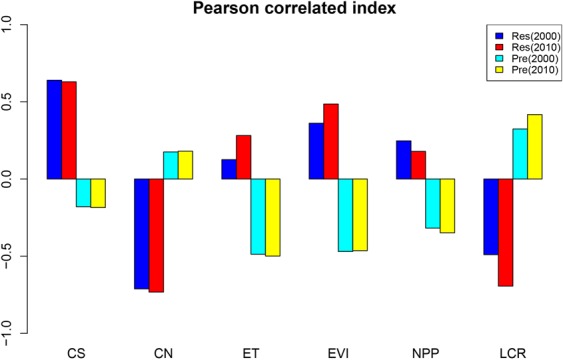
The correlated relationship between Response (Res) or Pressure (Pre) and ecological status index in 2000 and 2010. Figures were generated with R langauge program 3.1.3.

Three components of PSR framework for the 187 assessing units was shown by Fig. 4. The distribution pattern of pressure was similar. The range of pressure in 2000 year was from 0.01 to 0.96, which mainly located in the range from 0.04 to 0.15; yet the range in 2010 year was from 0.02 to 0.90, which mainly located in the range from 0.01 to 0.18. On average, the value of pressure increased from 0.145 in 2000 to 0.162 in 2010. The Pearson correlation analysis suggested that ecosystem pressure was significantly correlated with CN, LCR (local climate regulation) and the correlation intensity in 2010 is stronger than in 2000 (Fig. 5). The results suggested that the region was facing an increasing pressure with the rapid urbanization and increase of artificial surface resulted in degradation of water and heat regulating processes.

The distribution pattern of ecosystem status showed a little difference. In 2000, it mainly distributed between 0.22 to 0.42, while the main interval was from 0.22 to 0.34 in 2010. On average, the value of ecosystem status decrease from 0.378 in 2000 to 0.311 in 2010. With the urbanization process, the regional ecosystem status showed a general declining trend. The results confirmed that the deterioration of ecological process and function during two period.

The distribution pattern of ecosystem response for the two period was similar. More units located in the lower range from 0 to 0.13 in 2010, while more units distributed in the range from 0.13 to 0.25 in 2000. On average, the response decrease slightly from 0.096 to 0.087 during the two period. The Pearson correlation analysis suggested that ecosystem response was significantly correlated with carbon sequestration, ET, EVI,NPP and the correlation intensity in 2010 is stronger than in 2000 (Fig. 5). The indicator such as carbon sequestration, ET, EVI, NPP had the positive relationship with ecosystem response. These phenomenon suggested the ecological process and function can provided the important basis for ecosystem services.

In all, changes of three components of the PSR framework illustrated that the ecological risk was increasing with the urbanization process, facing greater pressure with lower ecosystem status and response.

### Ecological risk assessment

**Figure 6:**
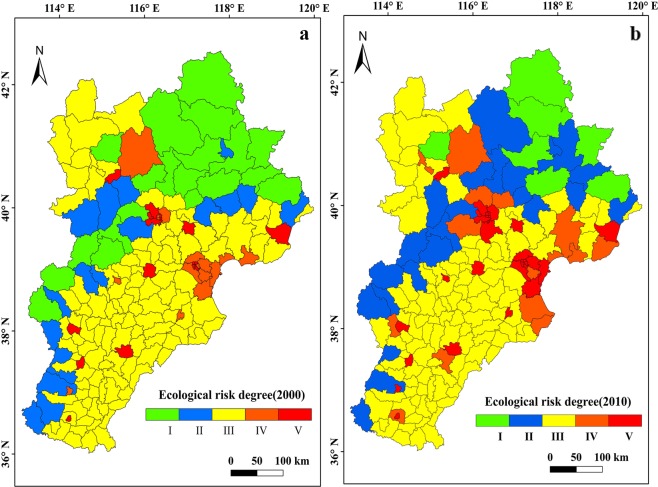
Ecological risk degree level. (**a**) Ecological risk degree level in 2000. (**b**) Ecological risk degree level in 2010. Map generated in ArcGIS 10.2.

Figure 6 illustrated the risk assessment results for the two period. The risk V to risk I region accounted for 10.70%, 10.15%, 60.96%, 8.56% and 9.63%, respectively, for the year of 2000, changing to 3.75%, 12.83%, 58.88%, 8.02% and 16.52%, respectively, in 2010. By comparison, the ratio of middle three risk degree II, III and IV was similar, only having small changes. The higher risk degree V increased 6.95%, while the lower risk degree I decrease 6.89% from 2000 to 2010. The results confirmed that the region was facing higher risk degree with the urbanization, indicating that the deterioration of ecosystem service and increase of ecosystem pressure.

**Figure 7:**
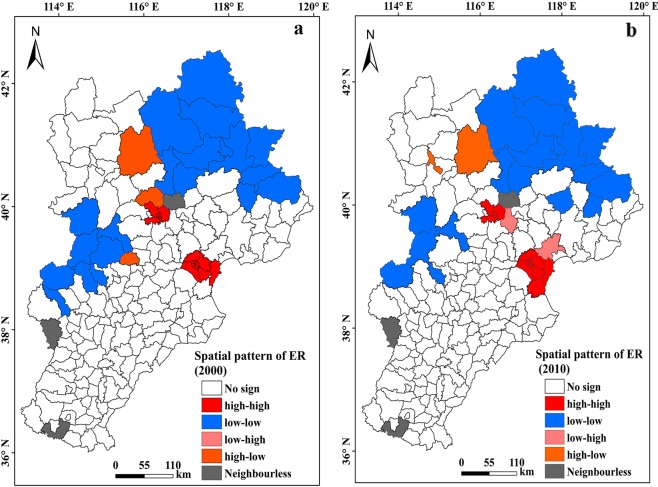
Spatial pattern analysis of ecological risk. (**a**) Spatial cluster of risk category in 2000. (**b**) Spatial cluster of risk category in 2010. Figure a and figure b generated in ArcGIS 10.2.

The spatial pattern about ecological risk degree level was showed by Fig. 7. Two main types including high value gathering field and low value gathering field existed in 2000. The higher gathering field owned the ratio of 9.85%, mainly distributed around the urban area of Beijing and Tianjin and scattered in southern urban region, while the lower gathering field possess a ratio of 10.69%, mainly distributed in the northern and western position of region. In 2010, the ratio of higher value gathering field increased by 0.53%, mainly happened in eastern of region. Moreover, the standardized value and spatial lag of high gathering field in 2010 year was higher than these value in 2000 year in the first quadrant. However, the ratio of lower gathering field declined by 2.12%, mainly located in the western position of region. The standardized value and spatial lag of lower gathering field in 2000 year was lower than these value in 2010 year in four quadrant. These results indicated that accompanied with the increase of impervious surface, the high ecological risk level in region represented increasing trend, while the low ecological risk level shown declining trend.

The coefficient for spatial auto-logistic model by MPLE (Maximum pseudo-likelihood estimation) was shown by Table 1. Based on the model figuration, the status played an important role for regulating ecological risk, and the response about ecosystem service also played a definite role for buffering risk. However, the pressure played a major role in leading the risk, and the spatial lag play an auxiliary role for enhancing the probability of risk. Based on the change of value about coefficient, only the coefficient for pressure showed increasing trend, that suggested that the ecosystem pressure have been increased in decade companied with the extension of impervious surface.

**Table 1:** The regression coefficient for spatial auto-logistic model.

Index	2000	2010
interception	1173.5	1092.1
pressure	483.613	501.127
status	−1792.87	−1683.87
response	−4.212	−4.079
Spatial lag of risk level	24.748	17.631
Residual test	0.01***	0.01***
AIC	10.00	17.34

## Discussion

The ecosystem service-oriented risk assessment framework combined with PSR model were applied to comprehensively evaluate the risk of regional ecosystem. Initially, the linkage and relationship between ecological mechanisms and ES that create the end products remains fuzzy^11,31^. However PSR could build the logical framework strengthening the linkage between ecological status and ecosystem service, partially could be made up for those shortcoming. Moreover, existing evaluation methods tend to focus on individual ES, that might resulted in a unbalanced assessment which fails to reveal the root mechanisms for consequent ecological risks for ES degradation^32^. Thus this study take four main types of ecosystem services as an integral system, that can narrow the knowledge gap between the impact on ecosystem service and complex ecosystem management. Although exponentially increasing studies of ES have advanced classification, evaluation and mapping techniques of ES, how to map and classify the risk degree level in the framework need further advance^33^. The classified method based on the distance about three components in PSR was analyzed in risk classification and relevant spatial risk regression model was applied.

The ecosystem inherent complexity needs to link assessment endpoints and ecosystem management, that needs to quantify service provision and possible adverse effects from socio-economical activities^34,35^. Moreover, ecological models have so much potential to deal with the problem that impedes ecologically related risk assessment. Thus, considering the ecosystem feature of complexity, two methods about machine learning was applied to simulate and evaluate risk assessment. Various index and evaluating model assisted the risk evaluation. First, the proper indicator for ecological process and function can be viewed as the prerequisites of ecosystem services, and it is necessary to take ecological indicator into the regional sustainable assessment^36,37^. Secondary, significant differences were ascertained among four type of ecosystem services (providing service; supporting service; regulating service; culture service) along the rural–urban gradient, particularly in urban environments where the values of these services were significantly lower than in rural and suburban area^14,16^. In the whole, theoretical and analytical frameworks about ecosystem service-oriented risk assessment have been further advancement in study.

## Methodology

### Study case

**Figure 8:**
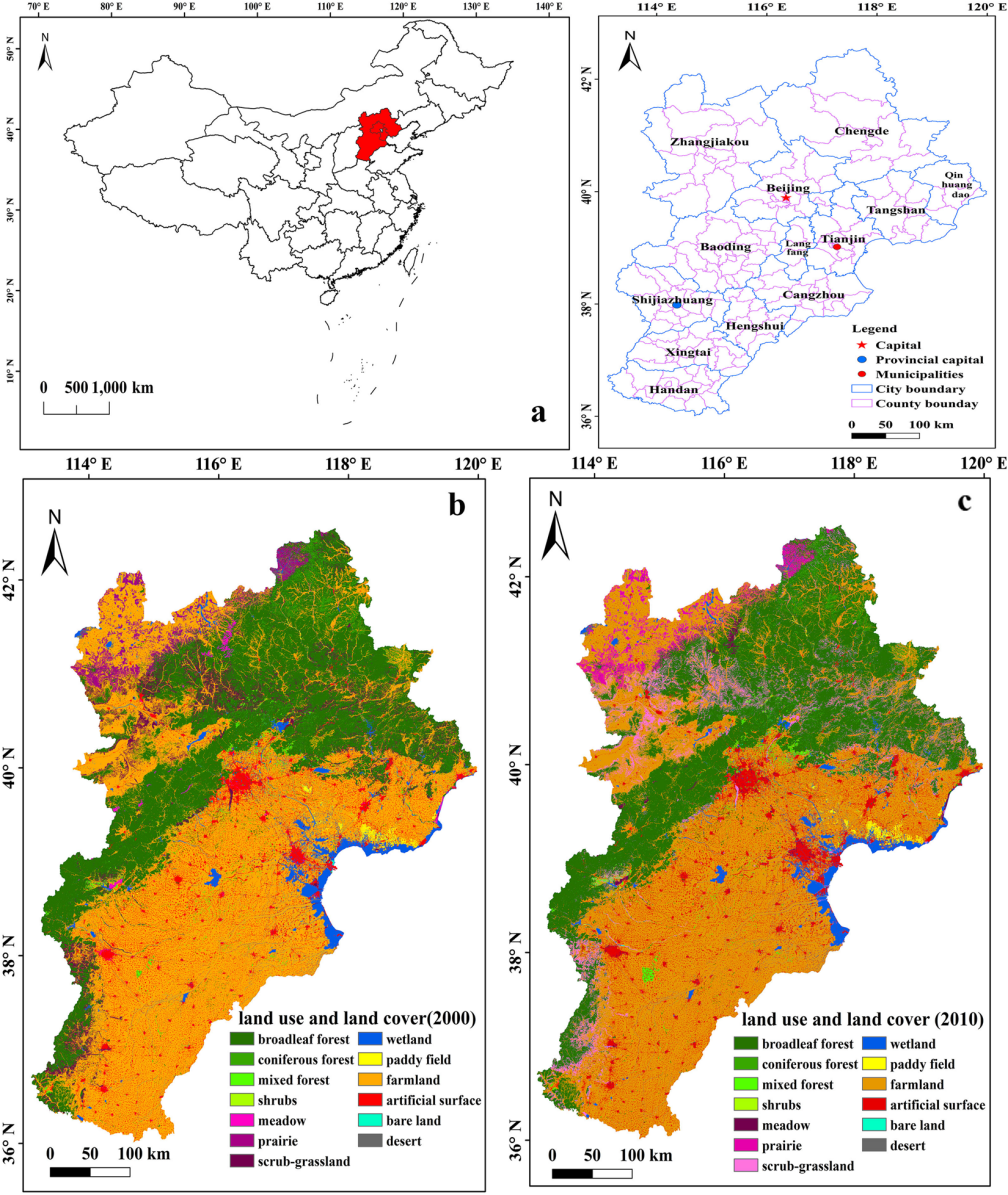
The study area of Beijing-Tianjin-Hebei urban agglomeration. (**a**) The location of Beijing-Tianjin-Hebei urban agglomeration in China. (**b**) Land cover and land use about Beijing-Tianjin-Hebei urban agglomeration in 2000. (**c**) Land cover and land use about Beijing-Tianjin-Hebei urban agglomeration in 2010. Map generated in ArcGIS 10.2.

The Beijing-Tianjin-Hebei urban agglomeration is one of the key fundamental development areas for China. It is located on the coast of the Bohai Sea (Fig. 8a) with a total area of 21.67 million ha, (113°27′–119°50′E, 35°03′–42°40′N) consists of Beijing, Tianjin, and Hebei province, accounting for 2.2% of the total land area of China^23^. The elevation in this region declines from the northwest to the southeast. This region is in the temperate zone, and the climate in summer is hot and rainy, while the winter is cold and dry. The average annual precipitation is approximately 538 mm and declines from the eastern coast to the western hinterland^23^. There are 13 cities in this region, thereof there including totally 187 assessing units. Beijing and Tianjin are megacities, with urban populations exceeding 10 million. Based on the comparison about the land use and land cover (LULC) of two period (Fig. 8b,c), the increase of the ratio of artificial surface reached to 1.03%, which located in the centre and east of region. The large-scale construction and development in region had brought a great deal of pressure on ecological environment.

### Methodology

#### Ecosystem service-oriented risk assessment framework

**Figure 9:**
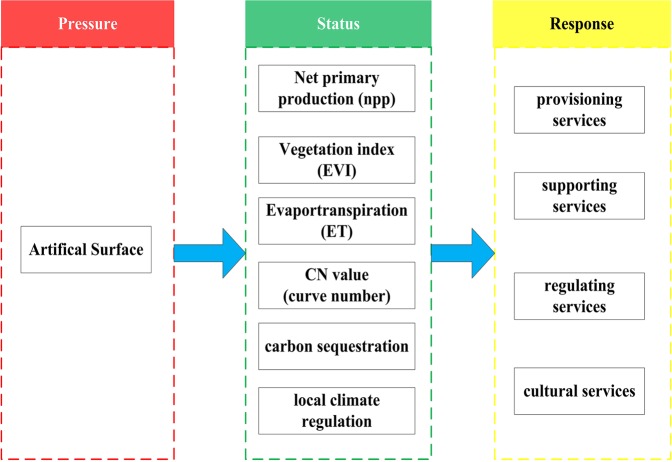
Ecosystem service-oriented risk assessment framework combined with PSR (pressure - status - response). Drawings were generated with Microsof Visio Professional 2007.

Regional ecosystem management problems and their solutions are often value-laden and subjective^38^. In order to comprehensively evaluating spatial-temporal interaction among artificial surface, ecological process or structure and ecosystem services, ecosystem service-oriented risk assessment framework combined with PSR (pressure-status-response) model was designed to evaluate the spatial-temporal impacts of urbanization on ecosystem (Fig. 9). The extension of artificial surface was viewed as the risk source, which represented by the urbanization ratio and reflected the pressure on regional ecosystem. Herein, night light data and the ratio of artificial surface was viewed as the pressure on ecosystem. A series of ecological index were properly selected, there were viewed as the status of ecosystem and represented the ecological structure, process and function. Finally, the change of ecosystem service can be taken as the response of regional ecosystem. The details on ecological status index, ecosystem service estimation, risk assessment and spatial analysis were illustrated in the following sections.

#### Index of ecosystem structure and process

A set of different proxy-based index were chosen to quantify the status of ecosystem^39^. Considering the structure, process and function of ecosystem and linkage with ecosystem services, we selected six index mainly includes NPP (net primary production), EVI (enhanced vegetation index), ET (evapotranspiration), CN value (curve number), carbon sequestration, and local climate regulation.

Among them, NNP defines the rate at which all plants in an ecosystem produce net useful chemical energy, which have the close linkage with provisioning services and supporting services. EVI are designed to provide consistent spatial and temporal comparisons of vegetation conditions, which has a direct with provisioning services, supporting services and regulating services. The indicator ‘carbon sequestration’ is chosen as an indicator of carbon cycle regulation, which is a positive indicator and contributes to global climate regulation and carbon supporting services^40^. So many types of ecosystem can act as carbon sinks and thus contribute to global carbon storage in a small but considerable amount. Evapotranspiration can be used to calculate regional water and energy balance, soil water status; hence, it provides key information for water regulating service and supporting service. CN (curve number) is selected for assessing the relevant capacity in hydrological process, that is directed with water regulating service and supporting services. Local climate regulation is presented by the ratio of temperature between each assessing unit and the mean value of vegetation in the same area, which can indicate the capacity of local climate regulation in the whole^41^.

#### Assessment of ecosystem services

To calculate ecosystem service values for each eco-region, we referred to Costanza’s ecosystem service values assessment model^42^ as follows:

1 $$ESV=\sum ({A}_{k}\,\ast \,V{C}_{k})$$

where *ESV* denotes the total values of ecosystem services while *A*_*k*_ and *VC*_*k*_ represent the area and value coefficient for proxy biome type ‘*k*’, respectively.

Referring to the revised ecosystem services assessment model of Xie *et al*.^43,44^ for different dominant LULC types in China, four main types of services including provisioning services, supporting services, regulating services and cultural services was obtained by the proxy map method, then the mean of each assessing units was counted.

#### Ecological risk assessment

Based on the PSR (Pressure-Status-Response) framework, the 12 selected indicators representing the pressure, state, and response indices of regional ecosystems was normalized in accordance with their attribute. Entropy method was applied to calculate the index weight about status and response part, then weight-index method was utilized to compute the comprehensive value. The radial basis neural network model combined with K-Means method was applied and simulated the ecological risk degree level. First, the five risk degree level base on the three index of PSR about 2000 year was optimally classified by 100 iterations in K-Means method. Then radial basis neural network model based on Gaussian Kernel function was simulated and tested based on three index of PSR and risk degree level about 2000 year. In study, the radial basis neural network model was constructed by 160 neurons, whose training accuracy and testing accuracy reached to 99% and 94.5%. Lastly the trained neural network model was used to predict the risk degree level about 2010 year.

Considering spatial heterogeneity in regional ecological risk assessment, Anselin local Moran’I was applied to analyze the local heterogeneity of ERD(ecological risk degree) and categorize the local neighboring relationship into four types(high-high, high-low, low-high, and low-low)^45^.

Pearson correlation analysis was used to quantify the relationship between ecological pressure, ecological response and ecological status index. Spatial auto-logistic model combined with maximum pseudo-likelihood estimator was utilized to fit for a logit regression model for risk probability. In study, different ecological risk degree level corresponded to risk probability, high risk degree level represents high probability, while low risk degree level represents low probability. Meanwhile, spatial dependent was assessed by spatial lag matrix within the distance of 40 kilometers. The function about model was as shown by Fun.2.

2 $${p}_{i}=P({y}_{i}=1||{W}_{{y}_{i}})=\frac{{e}^{\alpha +{X}_{i}^{\text{'}}\beta +r{W}_{{y}_{i}}}}{1+{e}^{\alpha +{X}_{i}^{\text{'}}\beta +r{W}_{{y}_{i}}}}$$

where *β* is a vector of parameters for exogenous variables, there included as the matrix consisted of pressure, status and response of ecosystem, *γ* is a scalar parameter for the spatial lag of *y* and ***W*** is a connectivity matrix. The spatial regression model performance was judged by pseudo *R*^2^ and Akaike Information Criterion (AIC).

### Data collection and analysis

The data of land use and land cover (LULC) were obtained from the project of national decade of remote sensing^35^. Landsat Thematic Mapper (TM) images for the years 2000 and 2010, aggregated into six main types, were collected and further divided into thirteen land types, forest (broadleaf forest, coniferous forest, mixed forest, and shrubs), grassland(meadow, prairie, scrub-grassland), wetland, farmland (paddy field, dry land), artificial surface, bare land and desert. The land use and cover in 2000 and 2010 years was showed by Fig. 8b,c. Based on the classification of land cover, the ratio of artificial surface was counted in each unit.

MOD17A3 (Terra/MODIS Net Primary Production Yearly), MOD16A3 (MODIS Global Evapotranspiration production yearly), MOD13A3 (Vegetation Indices Monthly) and MOD11A3 (MODIS temperature production Land Surface Temperature and Emissivity 8-Day) were acquired from EARTHDATA of NASA (http://ladswed.nascom.nasa.gov), and the resolution is 1 kilometers. Thereof, the yearly mean value was counted based on 12 images about each month; the mean temperature was calculated base on 14 images from June to September in summer, then local climate regulation index was counted. The land cover map and soils map was used as input for calculating curve number, which referring to a USDA-NRCS technical report^46^, using advanced implementations by Whitford *et al*.^47^ and Tratalos *et al*.^48^. The carbon sequestration capacity values of different land use types was derived from exiting studies^40,48^.

Classification of urbanization was ascertained using nighttime light imagery data (http://ngdc.noaa.gov/eog/dmsp.html)^49^. Recorded digital numbers (DNs) of nighttime light imagery data was applied to develop an index of urban sprawl for 2000 (DN2000) and 2010 (DN2010). Higher DN values represent higher levels of urbanization and the much stronger intensity of human activity. These measurements were used to divide the study area into three different levels of urban-rural gradient: developed urban, suburban, and rural. The classified rule of urban area is that DN value were more than 50 both in 2000 and in 2010, the classified rule of suburban area is that DN value was more than 50 in 2010 while less than 50 in 2000; the rural area is that DN value were less than 50 both in 2000 and in 2010. Finally, the urban-rural gradient for two period was analyzed based on the statistic of ecological status and ecosystem services. Considering the region locating in range (113°27′–119°50′E, 35°03′–42°40′N), thus there images in study were uniform projected to the Universal Transverse Mercator project system (datum WGS84, UTM Zone N50).

## Conclusion

This study contributes to a comprehensive understanding of how ecosystem service was influenced by artificial surface. Initially, the present study demonstrated the complexity of ecological index and ecosystem services performance along the urban–rural gradient of Beijing-Tianjin-Hebei urban agglomeration and thus adds to the understanding of both artificial surface and ecosystem change in an increasingly human-dominated world. Then a series of ecological index was selected and demonstrated that have a strong relationship with response and pressure (the ratio of artificial surface) by Pearson ‘correlated index. The PSR (Pressure-Status-Response) framework combining ecosystem service oriented risk assessment was established to comprehensively carry out risk assessment and classify risk degree. In total, ecosystem services was decreased and the ratio of artificial surface was increased that brought about ecological risk in rapid urbanization. As ecosystem service can act as a bridge between natural environment and social economy, incorporating this framework into resilience management in regional ecosystem could improve the understanding of the changes that urbanization brings to regional ecosystem, which is meaningful to prioritize strategies that can minimize the impacts of urbanization.

## Data Availability

The modeling data are available from the authors upon request.
